# Effect of CPAP treatment on subjective cognitive decline in patients with mild obstructive sleep apnea syndrome

**DOI:** 10.1007/s41105-025-00620-w

**Published:** 2025-11-24

**Authors:** Liang Tan, Xin Zhang, Zhijian Fu, Chunyao Dong, Chang Zhou, Caiyun Hou, Yuanyuan Lin, Xiaolei Wang, Li Li, Jin Zhou

**Affiliations:** 1 Department of Neurology, The first people’s hospital of Shenyang, Shenyang, China; 2 Department of Neurosurgery, The first people’s hospital of Shenyang, Shenyang, China

**Keywords:** Continuous positive airway pressure, Aerobic exercise, Obstructive sleep apnea syndrome, Subjective cognitive decline, Cognitive function

## Abstract

There is no consensus on whether to use continuous positive airway pressure (CPAP) treatment for mild obstructive sleep apnea syndrome (OSAS). Our study aims to observe the efficacy and necessity of using CPAP treatment in mild OSAS patients complicated with subjective cognitive decline (SCD). From August 2021 to July 2022, patients with mild OSAS and SCD were randomized to the control group or the CPAP group, and followed up for 3 months. The primary results were evaluated using subjective cognitive decline questionnaire (SCD-Q), the Huashan version of the Auditory Verbal Learning Test (AVLT-H), and animal fluency test (AFT), which were tested at admission and three months after treatment, respectively. And plasma Aβ1–40 and Aβ1–42 levels were also measured at admission and 3 months after treatment. A total of 110 patients completed the final study, with 55 in the control group and 55 in the CPAP group. No significant differences in demographic data, Laboratory tests, AHI index, SCD-Q, AVLT-H, and AFT scores between the two groups before treatment. The SCD-Q score of patients in CPAP group significantly decreased after 3 months of treatment and was significantly lower than that of control group (*P*<0.05). And the immediate memory score of AVLT-H in the treatment group was significantly increased after 3 months of treatment (*P* < 0.05). The Aβ1–42/Aβ1–40 ratio in the treatment group significantly decreased after 3 months of treatment, and was significantly lower than that of control group (*P*<0.05). Furthermore, there was linear correlation between SCD-Q score and Aβ1–42/Aβ1–40 ratio in all patients after 3 months of treatment (*P*<0.001, *r*=0.584). Our findings suggested that CPAP treatment can significantly decrease the Aβ1–42/Aβ1–40 ratio, and improve SCD, which may play an important role in the prevention of dementia, but further research are warranted.

**Supplementary Information:**

The online version contains supplementary material available at 10.1007/s41105-025-00620-w.

## Introduction

Obstructive sleep apnea syndrome (OSAS) is a common sleep disorder characterized with recurrent nocturnal apnea and intermittent hypoxia [1]. OSAS not only affects daily life, but also is related to dementia, stroke and other diseases, which seriously threatens human health [2, 3]. Continuous positive airway pressure (CPAP) is currently recognized as the most effective treatment method [4]. However, the compliance of CPAP is relatively low, especially for mild OSAS patients defined as the apnea–hypopnea index (AHI) between 5 and 15, they are often overlooked by their families due to the lack of obvious symptoms [5]. Studies have found that mild OSAS is very common in the community, with approximately 30–40% of the population suffering from mild OSAS [6, 7]. However, it is still debatable whether to use CPAP treatment for mild OSAS patients or not. And there are few research reports on whether CPAP treatment has therapeutic significance for mild OSAS patients with other complications.

With the increasingly trend of population aging, the number of people with cognitive impairment in China is growing rapidly. According to their severity, cognitive impairment is divided into SCD, mild cognitive impairment (MCI), and dementia. Cognitive impairment usually has an insidious onset and progresses slowly. As the disease progresses, treatment will become increasingly difficult [8]. MCI is an intermediate state between normal cognition and dementia, with a higher likelihood of transitioning to dementia [9] Therefore, interventions earlier than MCI may have better results. SCD, which refers to the deterioration of self-reported memory or more frequent loss or confusion of experiences, is a decrease in cognitive ability perceived subjectively by individuals, but without abnormalities detected by objective neuropsychological scales [10]. During 2015 to 2020, the prevalence of SCD in adults aged ≥ 45 years was 9.6% [11]. Previous studies have shown that OSAS is closely related to cognitive impairment and early dementia [12–14]. And SCD represents a risk stage which indicates the early stages of dementia and is an ideal stage for early intervention [15]. To date, there is still relatively little attention paid to mild OSAS, and there is no report on whether early CPAP treatment can improve SCD in patients with mild OSAS. Therefore, our study aims to analyze the improvement of cognitive function in mild OSA patients with concomitant SCD after receiving 3 months of CPAP treatment.

## Methods

### Trail design and participants

A randomized controlled parallel-group prospective study was designed. Patients diagnosed with mild OSAS and SCD were randomized into the two groups: the CPAP + aerobic exercise group which we defined as CPAP group and aerobic exercise group which we defined as control group. Randomization was performed using a computer-generated automated program including block randomization with a block size of 5.

Diagnostic criteria for SCD are based on

Inclusion criteria includes: (1) mild OSAS patients with AHI scores of 5 to 15; (2) meeting the diagnostic criteria for Subjective Cognitive Decline Proposed by Jessen in 2014 and the Chinese Association for Preclinical Alzheimer’s Disease [16] (Supplementary Table); (3) age ≥ 18 years; (4) The scores of the Simplified Mini-Mental State Examination and the Montreal Cognitive Assessment Scale are within the normal range; (5) without engaging in regular exercise in the past six months; (6) risk assessment was conducted before receiving treatment, and informed consent forms were signed by patients and their families. Exclusion criteria includes: (1) patients with other sleep disorders; (2) patients with diseases such as pulmonary bullae, hemoptysis, or pneumothorax who cannot use non-invasive ventilators; (3) patients with severe physical activity disorders; (4) having a history of mental illness or a family history of mental illness; (5) alcohol or drug dependent individuals; (6) unable to cooperate with survey respondents.

The survey was approved by the Ethics Committee of the First People’s Hospital of Shenyang, and was conducted in accordance with the Declaration of Helsinki (number: 2023SYKYPZ32). Written informed consent was provided by all participants or their legal representatives.

### Polysomnography and CPAP

Alice 4 multi-channel polysomnography produced in American company Respironics was conducted to assess the AHI score in all patients. Within 24 h before the examination, sleeping pills, alcohol, tea, or coffee are prohibited. The 5 ≤ AHI ≤ 15 times/hour is defined as mild OSAS.

CPAP ventilator (Philips, Respironics Inc, CNX700S17) will be used for pressure titration in CPAP group. The positive airway pressure should be adjusted until obstructive respiratory events such as apnea, hypopnea, respiratory effort related micro awakening, and snoring are eliminated. Based on this pressure, CPAP treatment will be carried out at home for no less than 5 h per night. The specialist will conduct regular follow-up and adjust parameters if necessary, and CPAP treatment will last for 3 months. To improve compliance, we assigned several follow-up nurses, and they would send text messages to patients at 7 p.m. every day to remind patients to complete the CPAP treatment in time. If there is no response, they wound also call to remind patients. In addition, standard out-clinic follow-up consultations are organized every month.

### Interventions

The CPAP group was given continuous positive pressure non-invasive ventilation during sleep, and patients were required to exercise for 45–60 min three times a week, including aerobics, square dancing, swimming, slow walking or cycling. Patients in control group were only required to exercise for a duration of 45–60 min three times a week, including aerobics, square dancing, swimming, slow walking, or cycling. And patients in both two groups are required to eat high fiber, low sodium and high protein foods every day.

### Assessments and outcomes

Blood samples were collected after fasting for at last 8 h within 24 h of randomization. Plasma Aβ1–40 and Aβ1–42 levels were detected at baseline and 3 months after treatment, respectively. And the automatically calculated Aβ1–42/Aβ1–40 ratio was collected.

At the time of patient randomization, and 3 months after treatment, the following questionnaire scores were performed: (1) Subjective Cognitive Decline Questionnaire (SCD-Q): The total score was 9 points, with a higher score indicating a higher probability of cognitive impairment, with a threshold of 5 points; (2) The Huashan version of the Auditory Verbal Learning Test (AVLT-H) is a vocabulary of 12 words that is repeated 3 times. The memory function of the subjects is evaluated based on the number and accuracy of the memorized words; (3) Animal fluency test (AFT): Participants are required to provide as many examples as possible in the animal category within 1 min to evaluate their language function status.

### Statistical analysis

Independent group comparisons are conducted using t-tests, while paired group comparisons are conducted using paired sample t-tests. And if the econometric data does not satisfy normality, it would be represented by the median (quartile). The Mann Whitney U test is used for independent group comparisons, and the Wilcoxon signed rank test is used for paired group comparisons. Categorical variables were described by frequencies with percentages, and the χ2 test was used to examine differences. The correlation between the two variables was analyzed by Pearson correlation analysis. For all the analyses, *p*<0.05 was regarded statistically significant. Statistical analyses were performed using the SPSS program (Version 26.0, IBM Statistics).

## Results

**Fig. 1:**
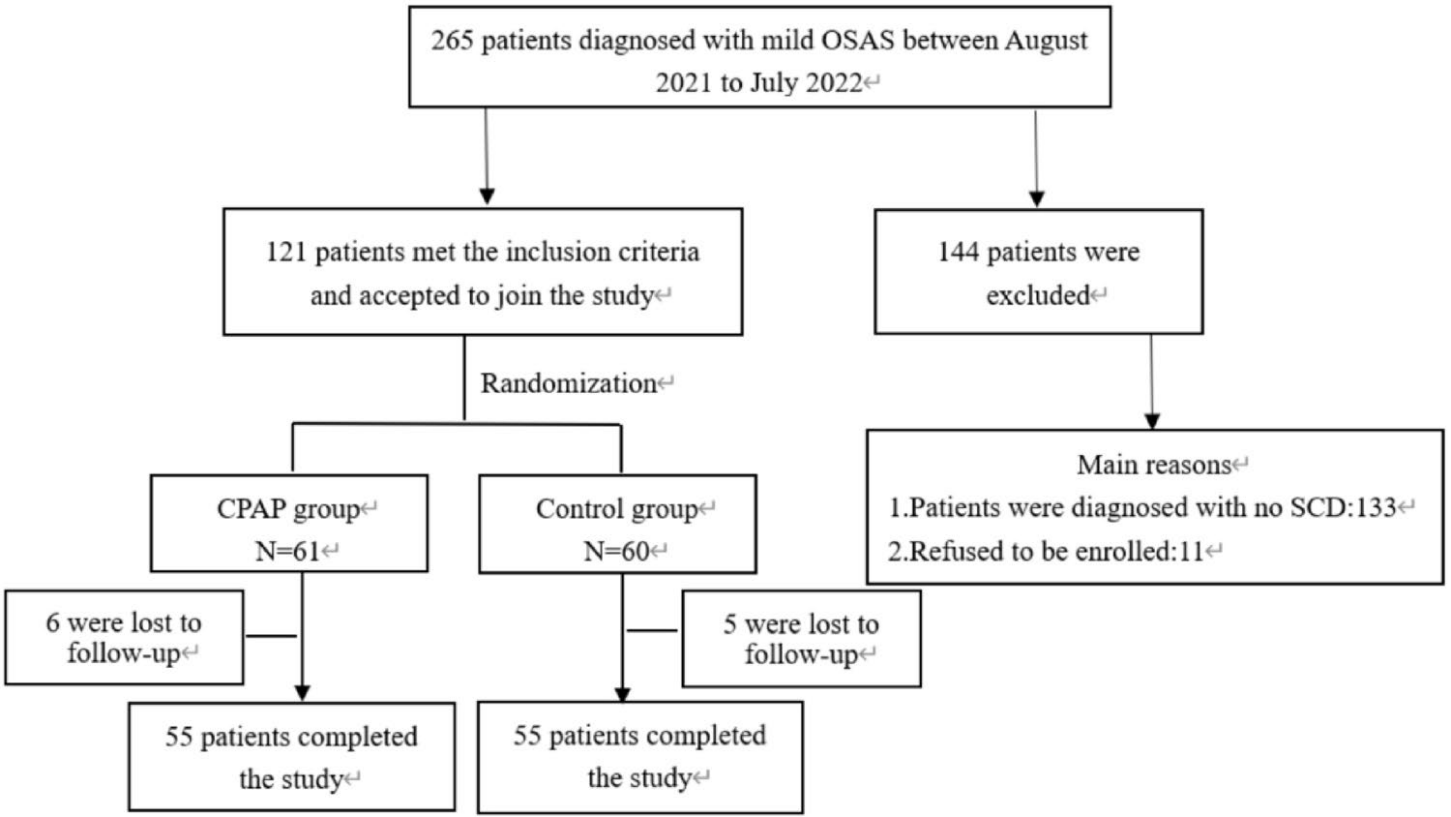
Study recruitment profile

From August 2021 to July 2022, we collected a total of 265 patients diagnosed with mild OSAS (5 ≤ AHI ≤ 15) in the First People’s Hospital of Shenyang. Among the 265 patients, 132 (49.8%) were diagnosed with SCD, accounting for approximately 50% in patients with mild OSAS, and 11 of which refused to be enrolled. As a result, 121 mild OSAS patients were ultimately enrolled and randomized CPAP group and control group. After 3 months of follow-up, a total of 110 patients completed the final study. Enrollment and randomization of the participants were shown in Fig. 1.

**Fig. 2:**
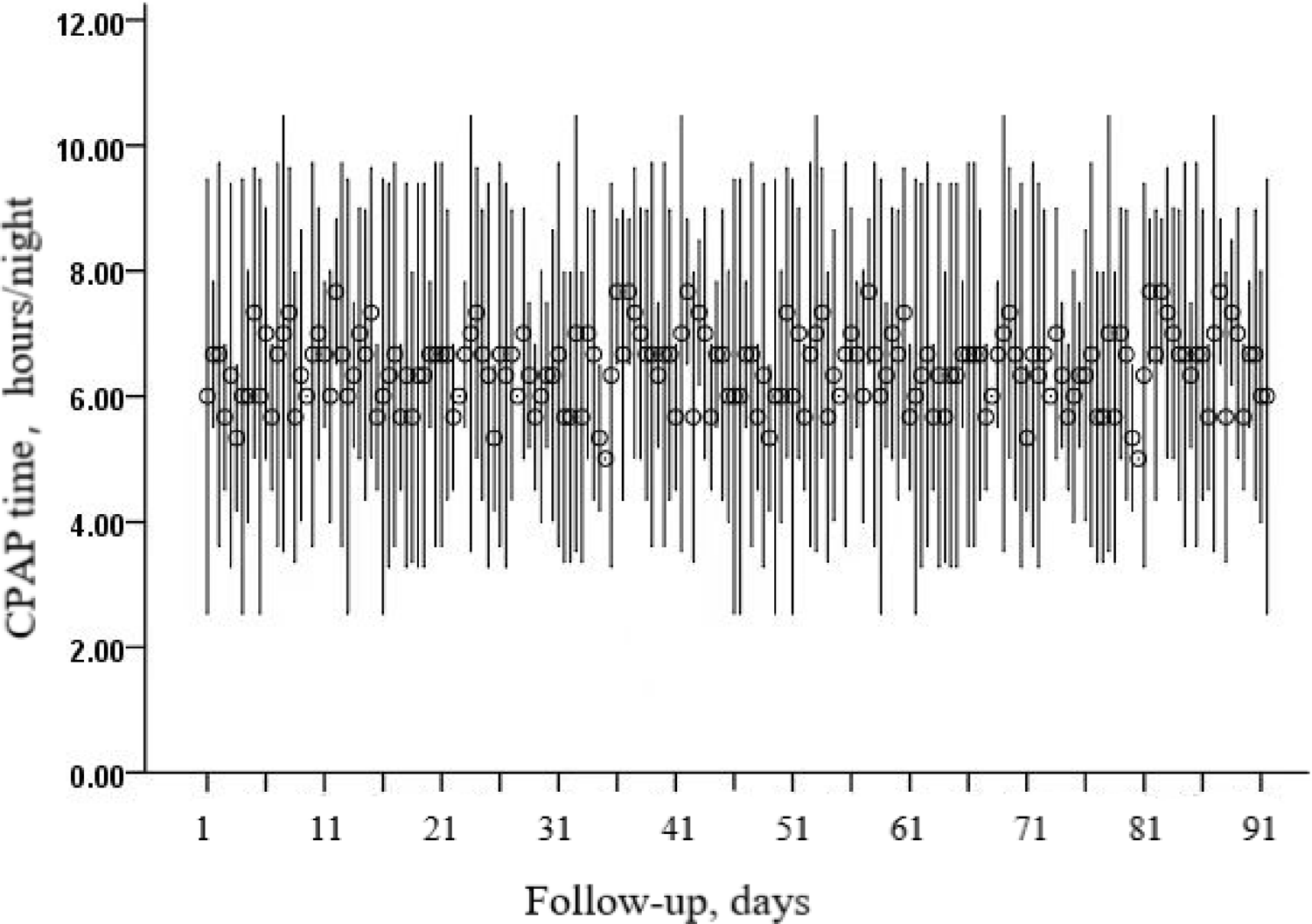
CPAP frequency and time during follow-up in CPAP group

The demographic data and clinical information are shown in Table 1. 55 patients were in the CPAP group and control group, respectively. No significant differences in age, sex, years of education, BMI, hypertension, smoking, drinking, cardiovascular disease, diabetes, and atrial fibrillation at admission was found between the two groups. Laboratory indicators at admission were also not statistically significant between the two groups. There is no statistically significant difference in the AHI index, the MMSE scores, and the MoCA scores between the two groups, indicating comparability. As shown in Fig. 2, the CPAP frequency during the 3 months of follow-up (mean ± SD) was 6.42 ± 0.55 days in the CPAP groups. And the CPAP time per night during the 3 months of follow-up was 6.47 ± 0.84 h in the CPAP groups.

**Table 1:** Basic characteristics of all patients between the two groups

Variables*	Control group (*n* = 55)	CPAP group (*n* = 55)	χ^2^/t	*P*
Sex (male)	32 (58.2)	39 (70.9)	1.947	0.163
Age (year)	64.95 ± 9.63	63.64 ± 11.09	0.661	0.510
Years of education (year)	9.49 ± 3.24	9.28 ± 3.09	1.064	0.336
BMI (kg/m^2^)	27.65 ± 2.54	27.83 ± 2.66	0.356	0.628
Hypertension (n, %)	16 (29.1)	18 (32.7)	0.345	0.557
Current smoking (n, %)	17 (30.9)	18 (32.7)	0.042	0.838
Current drinking (n, %)	6 (10.9)	6 (10.9)	0.000	1.000
Cardiovascular disease (n, %)	10 (18.2)	11 (20.0)	0.059	0.808
Diabetes (n, %)	20 (36.5)	26 (47.3)	1.345	0.246
Atrial fibrillation (n, %)	5 (9.1)	3 (5.5)	0.135	0.714
Uric acid, µmol/L	310.95 ± 89.19	320.64 ± 156.6	−0.399	0.691
HDL-cholesterol, mmol/L	1.12 ± 0.32	2.96 ± 13.62	−0.998	0.321
Triglyceride, mmol/L	1.64 ± 0.99	1.65 ± 0.77	−0.068	0.946
Total cholesterol, mmol/L	4.88 ± 1.49	4.72 ± 1.13	0.659	0.511
LDL-cholesterol, mmol/L	3.08 ± 1.28	3.07 ± 0.99	0.038	0.970
Glycated hemoglobin, %	6.65 ± 1.76	6.96 ± 1.68	−0.921	0.359
Hemoglobin, g/L	133.71 ± 24.03	139.96 ± 19.88	−1.487	0.140
HCY, µmol/L	66.53 ± 6.20	67.68 ± 7.94	−0.850	0.397
AHI score	9.82 ± 3.32	9.71 ± 3.01	0.180	0.857
MMSE score	27.54 ± 1.95	27.23 ± 2.07	0.808	0.421
MoCA score	27.79 ± 1.76	27.61 ± 1.89	0.517	0.606

HDL, high-density lipoprotein; LDL, low-density lipoprotein; HCY, homocysteine; AHI, apnea–hypopnea index; MMSE, Mini-Mental State Examination; MoCA, Montreal Cognitive Assessment Scale

*Continuous variables are expressed as mean ± standard deviation or median (interquartile range). Categorical variables are expressed as frequency (%)

There was no significant difference in SCD-Q scores between the two groups at baseline (*P* > 0.05); Compared with before treatment, the SCD-Q score of patients in the CPAP group significantly decreased after 3 months of treatment (*P* < 0.05), while the SCD-Q score in the control group did not decrease significantly (*P* > 0.05). And the SCD-Q score of patients in the CPAP group was significantly lower than those in the control group. There was no significant difference in AFT scores between the two groups of patients at baseline (*P* > 0.05); Compared with before treatment, after 3 months of treatment, the AFT scores of both groups of patients did not increase significantly (*P* > 0.05), as shown in Table 2.

**Table 2:** Outcomes of SCD-Q score and AFT score of all patients

		CPAP group (*n* = 55)	Control group (*n* = 55)	t/χ^2^	*P*
SCD-Q score	Before treatment	6.27 ± 1.33	6.16 ± 1.24	−0.443	0.659
	3 months after treatment	4.73 ± 1.46*	6.11 ± 1.23	5.376	< 0.001^#^
AFT score	Before treatment	16.18 ± 2.27	16.31 ± 1.98	0.313	0.755
	3 months after treatment	17.13 ± 2.40	16.35 ± 1.99	−1.860	0.066

SCD-Q, Subjective Cognitive Decline Questionnaire; AFT, Animal fluency test; CPAP, Continuous positive airway pressure; Compared to before treatment, *P < 0.05;Compared with the control group, ^#^*P* < 0.05

There was no significant difference in AVLT-H scores between the two groups of patients at baseline (*P* > 0.05). After 3 months of treatment, the immediate memory score of AVLT-H in the treatment group was significantly increased (*P* < 0.05), while the short delayed memory and long delayed memory scores were not significantly increased (*P* > 0.05). Furthermore, the AVLT-H scores in the control group were not significantly increased (*P* > 0.05), as shown in Table 3.

**Table 3:** Comparison of AVLT-H scores between two groups

Group	AVLT-H	Before treatment	3 months after treatment
CPAP group	Immediate memory	13.78 ± 2.71	17.65 ± 2.88*
	Short delayed memory	4.31 ± 0.96	5.42 ± 1.18
	Long delayed memory	4.05 ± 1.48	5.18 ± 1.59
Control group	Immediate memory	13.62 ± 2.67	15.07 ± 2.58
	Short delayed memory	4.24 ± 1.15	4.98 ± 1.3
	Long delayed memory	3.87 ± 1.49	4.67 ± 1.55

AVLT-H, The Huashan version of the Auditory Verbal Learning Test; CPAP, Continuous positive airway pressure;

Compared to before treatment, *P < 0.05

**Fig. 3:**
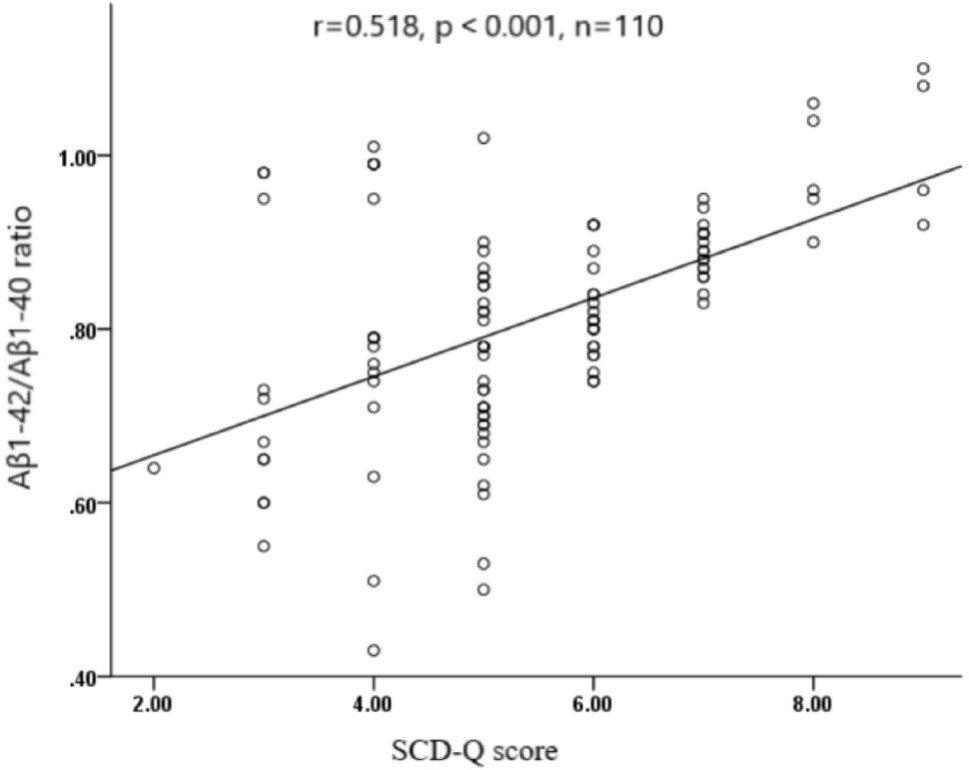
Linear correlation between Aβ1–42/Aβ1–40 ratio and SCD-Q score after 3 months of treatment

The levels of plasma Aβ1–40 and Aβ1–42 between two groups were shown in Table 4. And there was no significant difference in the Aβ1–42/Aβ1–40 ratio between the two groups at baseline (*P* > 0.05). We observed a significant reduction in Aβ1–42/Aβ1–40 ratio in CPAP group after 3 months of treatment (*P* < 0.05), but there was no significant change in control group (*P* > 0.05), as shown in Table 5. Figure 3 presents linear correlation between SCD-Q score and Aβ1–42/Aβ1–40 ratio in all patients after 3 months of treatment (*r* = 0.584, *p*<0.001). The decrease in the Aβ1–42/Aβ1–40 ratio is positively correlated with the decrease in SCD-Q score, as shown in Fig. 3.

**Table 4:** The levels of plasma Aβ1–40 and Aβ1–42 between two groups

	Time	CPAP group (*n* = 55)	Control group (*n* = 55)	t/χ^2^	*P*
Aβ1–42	Before treatment	106.23 ± 9.12	102.51 ± 8.13	2.058	0.126
	3 months after treatment	85.45 ± 7.61*	102.11 ± 7.69	11.420	< 0.001^#^
Aβ1–40	Before treatment	127.99 ± 9.79	120.60 ± 9.18	2.084	0.098
	3 months after treatment	108.16 ± 8.65*	123.02 ± 8.92	8.869	< 0.001^#^

CPAP, Continuous positive airway pressure; Compared to before treatment, *P < 0.05; Compared with the control group, ^#^*P* < 0.05

**Table 5:** Comparison of Aβ1–42/Aβ1–40 ratio between two groups

		CPAP group (*n* = 55)	Control group (*n* = 55)	t/χ^2^	*P*
Aβ1–42/Aβ1–40 ratio	Before treatment	0.83 ± 0.12	0.85 ± 0.13	1.092	0.277
	3 months after treatment	0.79 ± 0.12	0.83 ± 0.12	0.277	0.048*

CPAP, Continuous positive airway pressure; Compared with the control group, *P < 0.05

## Discussion

Our study found that CPAP combined with aerobic exercise treatment had a positive impact on SCD in patients with mild OSAS, and it can also improve immediate memory. Furthermore, the Aβ1–42/Aβ1–40 ratio of patients in the CPAP group significantly decreased after 3 months of treatment. Interestingly, there was a linear correlation between SCD-Q score and Aβ1–42/Aβ1–40 ratio after 3 months of treatment. These results suggest that CPAP combined with aerobic exercise improved SCD, and the Aβ1–42/Aβ1–40 ratio during treatment may play a positive role in this process.

OSAS can cause damage to multiple system organs, including cardiovascular system diseases, stroke and depression, etc [17]. And there is high proportion of subjects with DM in our study, which might be related to being older and being overweight. Research has shown that the decrease in minimum blood oxygen saturation at night in OSAS patients is closely related to cognitive impairment in memory, attention, and language [18]. And OSAS can increase the risk of Alzheimer’s disease [2]. The development of Alzheimer’s disease can generally be divided into subjective cognitive decline, mild cognitive impairment, and dementia. And SCD patients include a variety of individuals, including those who will return to normal, those with Alzheimer’s pathology, and those with non-Alzheimer’s pathology [19]. A meta-analysis found that the incidence of mild cognitive impairment and dementia in patients with SCD after 4 years was 27% and 14%, respectively [16]. SCD might be the earliest clinical manifestation of Alzheimer’s disease, and this stage provides an important time window for delaying or even preventing the progression of Alzheimer’s disease [20]. In our study, nearly 50% of mild OSAS patients have SCD. Just as our research has found, after 3 months of CPAP combined with aerobic exercise treatment, the SCD-Q score significantly improved, which may slow down the progression of Alzheimer’s disease.

CPAP is the preferred treatment method for adult OSAS patients [21, 22]. Previous study found that CPAP can reduce the risk of cardiovascular disease and improve prognosis in patients with OSAS [23]. Liu et al. [24]found CPAP can improve the decrease in white matter integrity caused by OSA, which may be the reason for cognitive and emotional improvement after CPAP treatment. Furthermore, Condoleo et al. [14] found that CPAP treatment was able to improve cognition and depressive symptoms in elderly patients suffering from moderate to severe OSAS. However, previous studies have mostly focused on the treatment of moderate to severe OSAS, with limited attention paid to the treatment of mild OSAS. Our study found that the incidence of SCD in patients with mild OSAS was 49%, and these patients were at risk of developing dementia, potentially endangering people’s health. Our study demonstrates that CPAP can effectively improve SCD. Therefore, for patients with mild OSAS, if combined with SCD, it is recommended to regularly apply CPAP to delay the progression of dementia and improve prognosis.

The deposition of Aβ protein in the cerebral cortex and hippocampus is one of the pathological characteristics of Alzheimer’s disease. It gradually aggregates to form soluble oligomers and eventually forms insoluble amyloid plaques [25]. There are two main types of Aβ protein: Aβ 1–40 and Aβ 1–42 [26]. Previous studies have shown that compared with the normal control group, the plasma levels of Aβ1–40 and Aβ1–42 in the mild cognitive impairment (MCI) group and the SCD group were elevated [27]. As our research has found, after 3 months of CPAP treatment, there was also a certain degree of decrease in plasma Aβ1–40 and Aβ1–42 levels, indicating improvement in cognitive function. With regard to Aβ1–42, a normalization of values in relation to Aβ1–40 has been established, namely the Aβ1–42/Aβ1–40 ratio [28]. Aβ1–42/Aβ1–40 ratio will improve the biomarker analytical variability, and will also improve early and differential AD diagnosis through a more accurate reflection of pathology [29]. Although there is a consensus on the reduced levels of Aβ 1–42 in the CSF of patients with AD, studies of plasma Aβ 1–42 levels were inconsistent. Chiu et al. [30] found that the plasma levels of Aβ 1–42 and the Aβ1–42/Aβ1–40 ratio tended to increase with the severity of the dementia. Our study also reached a consistent conclusion. Our study found that the Aβ1–42/Aβ1–40 ratio in the treatment group significantly decreased after 3 months of treatment, and the Aβ1–42/Aβ1–40 ratio in the treatment group was significantly lower than that in the control group after 3 months of intervention. Furthermore, there was linear correlation between SCD-Q score and Aβ1–42/Aβ1–40 ratio in all patients after 3 months of treatment. Therefore, it can be seen that CPAP treatment is beneficial for reducing the Aβ1–42/Aβ1–40 ratio, and reducing the incidence rate of SCD in mild OSAS patients.

Currently, there are relatively few mild OSAS patients who use CPAP for treatment. Do all mild OSAS patients not need to use CPAP treatment? Previous studies have shown a close relationship between OSAS and depression, and they found an amelioration of depression under CPAP-adherent patients as well [24]. Likewise, our research findings indicated that approximately half of mild OSAS patients were accompanied by SCD, which may further progress to dementia. The benefits we observed in patients with CPAP treatment, are important enough to be taken into account in the management of patients with mild OSAS, because our goal is not only to control patients’ symptoms but also to reduce the risk of dementia. And there may be better effects in memory and cognition after a longer period of CPAP treatment, which requires further research to confirm.

### Limitations

A limitation of our study is that our follow-up time was relatively short, and the improvement of SCD may also be related to changes in lifestyle habits and the improvement of daytime sleepiness symptoms. However, we were pleasantly surprised to find that certain dementia related indicators, such as Aβ1–42/Aβ1–40 ratio, improved in a beneficial direction through CPAP treatment, which also enhanced the reliability of our experiment. In the future, we will conduct longer follow-up to further explore.

## Conclusions

In conclusion, CPAP treatment can significantly decrease the Aβ1–42/Aβ1–40 ratio, and improve SCD, which may play an important role in the prevention of dementia. Furthermore, CPAP treatment may have a certain improvement effect on immediate memory, and there may be better effects in memory and cognition after a longer period of CPAP treatment, which requires further research to confirm.

## Supplementary Information

Below is the link to the electronic supplementary material.

Supplementary Material 1

## Data Availability

All data generated and analysed during the current study are included in this published article.
