# Supplementary material for: Effect of CPAP treatment on subjective cognitive decline in patients with mild obstructive sleep apnea syndrome
Source: Sleep Biol Rhythms. 2025 Nov 24;24(1):123–30. doi: 10.1007/s41105-025-00620-w (PMC12804546; doi:10.1007/s41105-025-00620-w)
Supplement: Supplementary file 1 — Supplementary Material 1 [file 41105_2025_620_MOESM1_ESM.docx]

**Supplementary Table**

**Diagnostic criteria for subjective cognitive decline:** **Expert Consensus on Preclinical SCD Diagnostic Procedures and Norms for AD in China (2020 Revision)**

(1) Sustained decline in perceived cognitive function compared to the previous normal state, independent of acute events.

(2) After adjusting for age, sex, and years of education, exhibited normal performance on standard cognitive tests (in contrast to those with MCI and predementia).

Both (1) and (2) must be present.

Exclusion criteria:

(1) MCI, prodromal dementia AD or dementia

(2) Cognitive decline explained by psychiatric or neurological disorders * (except AD), medical conditions, drug side effects, or substance abuse

* Exclusion criteria do not include symptoms of anxiety or depression without meeting the diagnostic criteria for the corresponding disorder. And MRI brain had been done in all patients at the time of enrollment. Only those without serious neurological disorders (such as Parkinson's disease), severe vascular events (such as stroke), or recent or distant history of severe traumatic brain injury were included in the study.
